# Quantitative Postural Evaluation in Orchestral Musicians (the OPErA Project): Protocol for a Cross-Sectional Study

**DOI:** 10.2196/81885

**Published:** 2026-06-22

**Authors:** Claudia Consentino, Marina Ramella, Sara Da Pian, Alessandro Gastaldelli, Pier Carlo Battain, Rosa Maria Converti

**Affiliations:** 1 Smart Homes, Assistive Technologies, and Occupational Therapy (DAT) Research Unit, IRCCS S Maria Nascente, Fondazione Don Carlo Gnocchi, Via Capecelatro 66, Milan, Italy, 39 0240308244

**Keywords:** posture, postural assessment, ergonomic assessment, musicians, rehabilitation, prevention

## Abstract

**Background:**

Musicians are at high risk for playing-related musculoskeletal disorders due to prolonged static postures and asymmetrical movements. Despite the prevalence of these disorders, objective ergonomic assessments in orchestral settings are limited.

**Objective:**

The primary aim of this study (the Orchestral Posture Ergonomic Assessment [OPErA] project) is to quantify postural deviations in professional orchestral musicians, with and without their instruments, and to investigate their association with pain and pain leading to performance limitation. Secondary aims include exploring demographic, clinical, and occupational factors associated with these outcomes.

**Methods:**

This cross-sectional study will enroll 250 professional musicians from Italian orchestras, excluding pianists. Postural assessments will be conducted in 2 phases—without and with the instrument—using the Physical Analyzer Portable, a device for calibrated photo acquisition. Anatomical landmarks will be marked with electrocardiogram electrodes to measure inclination, rotation, and asymmetries in the frontal and sagittal planes. Pain and disability will be evaluated using validated questionnaires (Quick Disabilities of the Arm, Shoulder and Hand [QuickDASH]; Modified Oswestry Disability Index; and Neck Disability Index). Statistical analysis will include descriptive statistics, 2-tailed *t* tests, chi-square tests, and regression models to explore associations between posture and pain.

**Results:**

The protocol was approved in November 2022 and funded in 2025. Data collection was conducted from May 2023 to April 2026. By the time of manuscript submission (February 2026), 240 of the target 250 participants had been enrolled. Data analysis is ongoing, and the results are expected to be published in July 2026.

**Conclusions:**

This study will provide quantitative insights into postural deviations and their relationships with pain and pain leading to performance limitation. The findings are expected to identify instrument-specific associated factors and compensatory behaviors, supporting the development of targeted ergonomic interventions and preventive strategies for musicians’ health.

## Introduction

### Background and Rationale

Movement is essential for musicians, enabling them to express creativity and connect with their audience. The integration of body and instrument creates an impression of effortless harmony, free from tension and pain. However, this idealized image often obscures the reality of musculoskeletal and neurological disorders that can affect musicians, limit performance, and, in some cases, end careers prematurely. Musicians often ignore pain and symptoms that need attention and rest [1].

Bernardino Ramazzini first described occupational diseases in musicians in 1713. Zaza et al [2] introduced the term playing-related musculoskeletal disorders (PRMDs) to describe musculoskeletal symptoms, such as pain, weakness, numbness, or loss of coordination, that are caused or exacerbated by playing a musical instrument and are severe enough to impair a musician’s ability to perform at their usual technical level. In this definition, a condition qualifies as a PRMD only when it interferes with musical performance, distinguishing it from symptoms that may occur in everyday life but do not affect playing. Zaza et al [2] further emphasized that PRMDs can become chronic and disabling, affecting musicians not only physically but also emotionally and socially [3]. Reviews report high prevalence rates of musculoskeletal disorders among musicians, with rates ranging from 57% to 68% for general issues, 9% to 68% for PRMDs, and 62% to 93% for one-time PRMDs [1,4,5]. Studies, such as those by Fishbein et al [6], show that many musicians, including 76% of International Conference of Symphony and Opera Musicians members, experience medical issues that compromise their performance.

Identifying risk factors for PRMDs is crucial for prevention [7]. Common factors include gender, age, professional status, years of experience, type of instrument, repertoire, technique, long practice hours, poor physical condition, muscle fatigue, lack of preventive behaviors, and previous injuries. Psychological stress factors, such as anxiety, depression, and perfectionism, also correlate with musculoskeletal disorders [1,4,8]. The weight of the instrument and playing posture can influence injury prevalence.

Research confirms that prolonged forced body positions increase the likelihood of musculoskeletal overload. Few studies focus on the impact of playing classical instruments on posture [9]. Musicians spend about 1300 hours per year in ergonomically unfavorable positions. Instruments requiring different playing positions and techniques have varying prevalence rates of musculoskeletal disorders [10]. A study among conservatory students found high rates of postural alterations without the instrument (66.2%) and nonoptimal posture with the instrument (73.4%). Asymmetrical postures, such as those required by the violin, viola, and flute, are significant associated factors [11].

Musicians playing with arms above the scapulohumeral plane are more prone to PRMDs in the upper limbs [12]. Addressing these conditions requires understanding the factors and implementing appropriate measures. Reviews on ergonomic assessment in orchestra musicians indicate a lack of publications on this topic [13]. More information is available on questionnaires assessing symptoms, such as the “standardized Nordic Musculoskeletal Questionnaire” [14] and the Disabilities of the Arm, Shoulder, and Hand [15].

Compensatory behaviors can be a significant associated factor linked to musculoskeletal disorders among musicians. Understanding and addressing these behaviors is essential for effective prevention and management.

### Objectives

The study aims are to investigate the frequency and types of postural changes, both while musicians are playing their instruments and when they are not, and evaluate whether these postural changes are associated with pain and pain leading to performance limitation. The study will also explore additional associated factors with postural changes and pain based on the demographic, clinical, and musical activity information obtained through the questionnaire.

Finally, it will explore how these postural issues affect the professional lives of musicians.

## Methods

### Study Design

The study design chosen for this project is cross-sectional.

#### Study Setting

The setting will not be standardized: postural assessments will be performed in the various sites of the participating orchestras. Image acquisition was conducted in private changing rooms provided by the hosting institutions (eg, theaters), following an explicit request from the head of Smart Homes, Assistive Technologies, and Occupational Therapy unit. Only the researcher responsible for image collection was present during the procedure; the physician supervising participant enrollment could enter the room only if required. Images were stored on a secure institutional server with restricted access. Raw photographs, which included participants’ faces, can be accessed exclusively by the medical members of the research team and the therapist involved in enrollment. The data collected will be processed and stored by Fondazione Don Carlo Gnocchi for the time necessary to achieve the purposes of the specific study, in compliance with the principle of data minimization and with legal obligations. After this period, the data will be retained for 10 years following the conclusion of the study. The key linking patient identity and study ID will be kept by the principal investigator in a protected file stored outside the digital database for the duration of the study and will be deleted at its completion.

#### Eligibility Criteria

Inclusion criteria were being a professional musician in an Italian orchestra, being aged >18 years, belonging to either sex, and having signed the informed consent form. The only exclusion criterion was having the piano as the primary instrument, owing to the logistical challenges associated with its size and transportability, which precluded standardized evaluation.

#### Interventions and Outcomes

Demographic, medical history, and musical activity data will be collected using a specifically designed questionnaire. This questionnaire will also gather information on rehearsal frequency and playing duration during the previous week, as these variables represent more stable and comparable exposure indicators across participants. Conversely, information regarding rehearsal conditions and playing duration on the day of assessment will not be collected, as these factors exhibit substantial day-to-day variability, cannot be standardized across orchestra settings, and are unlikely to exert a reproducible influence on static postural parameters.

The questionnaire used in this study is not a validated instrument; it was developed based on the research team’s clinical experience. It was administered in Italian. Participants were first asked to report any history of performance-limiting pain throughout their entire professional career. Subsequently, targeted questions evaluated the presence of pain and PPL during the preceding week, allowing the distinction between lifetime occurrence and recent symptoms.

The impact of painful symptoms during last week on professional and nonprofessional activities will be assessed using the “QuickDASH” scale for the upper limb disability [16], the “Modified Oswestry Low Back Disability Index” for low back pain [17], and the “Neck Disability Index” questionnaire for neck pain and cervicodorsal symptoms [18].

Postural evaluation will consist of 2 phases: phase 1 without the instrument in a sitting position and phase 2 with the musical instrument in a sitting position. To improve the precision of the examination during data acquisition, participants will be asked to remain shirtless if male or wearing only a bra if female. Disposable electrocardiogram electrodes will be applied to anatomical landmarks. These electrodes are used exclusively as anatomical markers and not for recording electrical activity. They consist of circular adhesive pads with a base diameter of approximately 30 mm to 40 mm and a small, raised apex of a few millimeters, which facilitates precise landmark identification. The following anatomical landmarks were identified: spinous process C7 (C7), right shoulder acromion (RS), left shoulder acromion (LS), right scapula inferior angle (RIA), left scapula inferior angle (LIA), spinous process L3 (L3), intergluteal line (IG), glabella (GL), and sternal notch (ST).

Participants were seated on a standardized stool (height: 47 cm; leg separation: 26.5 cm), except for the double bass players who used a chair provided by the orchestra without a backrest due to the instrument’s size and ergonomic requirements. The stool, the chair, and the camera were placed in fixed positions, which were marked on a mat included in the kit, and the instrument was calibrated according to these distances.

For photos without the instrument, participants kept their arms relaxed alongside the body. For photos with the instrument, hand placement followed the standard playing position (a position for string instruments). In both phases, the Physical Analyzer Portable will be used—a noninvasive, portable device that allows calibrated photo acquisition on the frontal and sagittal planes and subsequent data processing through dedicated software. This device is not formally validated but it is aligned with the principles of clinical posture assessment presented in the study by Fortin et al [19]. The following measurements will be taken with and without the musical instrument.

As far as the frontal plane is concerned, the following parameters will be measured: trunk inclination on the frontal plane: angle between C7 and IG, head-neck inclination: angle between ST and GL, right shoulder height: distance between RS and the horizontal line passing through C7, left shoulder height: distance between LS and the horizontal line passing through C7, right shoulder width: distance between RS and the vertical line passing through C7, left shoulder width: distance between LS and the vertical line passing through C7, right scapula–rachis distance: distance between RIA and the vertical line passing through C7, and left scapula–rachis distance: distance between LIA and the vertical line passing through C7.

As far as the sagittal plane is concerned, the following parameters will be measured: cervical arrow: distance between the vertical tangent to the apex of the dorsal kyphosis and C7, lumbar arrow: distance between the vertical tangent to the apex of the dorsal kyphosis and L3, right shoulder distance: distance between RS and the vertical line passing through C7, and left shoulder distance: distance between LS and the vertical line passing through C7. We will numerically quantify trunk rotation and right-left shoulder asymmetry in both conditions (with and without the instrument). Furthermore, we will compute all between-condition differences to assess postural changes attributable to instrument use. All distances are measured in millimeters, and all angles are measured in degrees.

Musical instruments will be classified into 2 main categories: symmetric and asymmetric [11], and the postural measures will be analyzed based on this categorization. Further exploratory analyses may also be conducted on specific instrument families.

#### Timeline

The project phases were implemented according to an indicative timeline spanning November 2022 to December 2024. The initial phase involved defining the clinical framework of the OPErA (Orchestral Posture Ergonomic Assessment) project and preparing the documentation for submission to the ethics committee. This was followed by orchestra recruitment, implementation of the study protocol, data collection and analysis, and the preparation of related reports.

#### Sample Size

A total of 250 participants will be enrolled. Regarding the primary objective of the study, which is to investigate the prevalence and types of postural alterations, it is estimated that—assuming a prevalence of 50%—a sample of 250 participants will allow for an estimation of the prevalence with a significance level of α=5% and a precision (semiwidth of the CI) of 6%, using the binomial model with normal approximation for the calculation of the CI.

Participant recruitment will be conducted following ethics committee approval and registration of the study on ClinicalTrials.gov, with an anticipated recruitment period of 30 months.

Participation in the study will be voluntary and will require written informed consent. All musical instruments will be eligible for inclusion and will be classified as either symmetrical or asymmetrical for analysis purposes.

#### Recruitment

Postural assessments will be performed at the various locations of the participating musicians’ orchestras.

### Data Collection, Management, and Analysis

All collected data (demographic, clinical, postural, and questionnaire data) will be managed to ensure participant confidentiality. Data collection will use Research Electronic Data Capture (REDCap; Vanderbilt University), hosted on a secure server at Fondazione Don Carlo Gnocchi. Each participant will be assigned a unique study ID. The key linking the ID to personal identity will be stored in a password-protected file, separate from the research database, accessible only to the principal investigator. All data will be pseudonymized for analysis.

Photographs taken for postural assessment are considered sensitive personal data. Raw images containing faces will be accessible only to designated medical personnel and the enrolling therapist directly involved in the study. In accordance with the approved ethical protocol, all raw photographs will be permanently deleted upon completion of data analysis. The pseudonymized postural measurement dataset and questionnaire responses will be retained for 10 years after study conclusion for archival purposes, as required by Italian regulation and the funding body, on the secure institutional servers.

### Statistical Methods

Statistical analysis will be performed using SPSS Statistics software (version 27.0; IBM Corp). The primary analysis will focus on within-subject comparisons of postural parameters measured in the 2 experimental conditions (without instrument vs with instrument). Continuous variables will be described using the mean and SD or the median and IQR based on their distribution. The Kolmogorov-Smirnov test will be used to assess the normality of the distributions. Categorical variables will be reported in terms of relative frequencies.

### Main Comparison: Postural Changes With and Without the Instrument

To address the primary objective of quantifying postural deviations induced by instrument playing, postural measurements obtained in the 2 conditions will be compared using paired statistical tests. Specifically, for continuous postural parameters such as shoulder height difference, cervical arrow, and scapula-rachis distance that are normally distributed, the 2-tailed paired *t* test will be used. For parameters deviating from normality, the Wilcoxon signed-rank test will be applied. These analyses will be conducted on the entire sample and subsequently stratified by instrument category (symmetric vs asymmetric) to elucidate instrument-specific effects. For each parameter, the magnitude of postural change will be calculated as the delta value, defined as the measurement obtained with the instrument minus that obtained without the instrument. These delta values constitute the primary measures of playing posture and will serve as key independent variables in subsequent regression models.

### Handling of Multiple Postural Measures

Given the multiplicity of postural parameters assessed, there exists an inherent risk of type I error inflation due to multiple comparisons. To mitigate this, several strategies will be implemented. Although all postural measures are considered exploratory, the analysis will prioritize a core set of clinically relevant parameters identified a priori based on the extant literature, including head-neck inclination, shoulder asymmetry, and lumbar arrow. As an alternative to multiple univariate comparisons, multivariate analysis of variance for repeated measures may be used to compare the overall postural profile between conditions, provided the underlying assumptions are satisfied. For exploratory analyses involving multiple univariate paired comparisons, conservative corrections such as the Bonferroni adjustment or false discovery rate control using the Benjamini-Hochberg procedure will be applied. Findings will be interpreted judiciously, and effect sizes, including Cohen *d* for paired samples, will be reported alongside *P* values to emphasize the magnitude of observed effects rather than relying solely on statistical significance.

### Handling of Missing Data

Minimal missing data are anticipated owing to the standardized data collection protocol. Nevertheless, any missing values in postural measurements, for instance, due to obscured anatomical landmarks in photographic acquisitions, will be systematically reviewed. Should data be determined to be missing completely at random, complete case analysis will be performed. If the proportion of missing data exceeds 5% for a key variable, multiple imputation techniques will be considered, provided the missing at random assumption is tenable. Missing data in questionnaire responses will be addressed in accordance with the scoring manuals of each respective instrument, including the QuickDASH and Neck Disability Index.

### Association With Pain and Disability (Secondary Aims)

To examine factors associated with the outcomes of pain and performance-limiting pain, logistic regression models will be used. The dependent variables will be the presence of pain during the preceding week and the presence of performance-limiting pain during the preceding week, both operationalized as binary outcomes. Independent variables will include demographic characteristics (eg, age and gender), occupational factors (eg, instrument type [classified as symmetric or asymmetric], years of professional experience, and practice hours), and postural delta values, which represent instrument-induced postural changes as described above.

To investigate factors associated with the severity of disability, as measured by the QuickDASH, Oswestry Disability Index, and Neck Disability Index scores, linear regression models (or quantile regression models, if model assumptions are violated) will be used, incorporating the same set of independent variables.

Variables achieving a *P* value of <.10 in univariate analyses will be entered into multivariable models to identify independent predictors. Results will be reported as odds ratios or regression coefficients with their corresponding 95% CIs. A *P* value of <.05 will be considered indicative of statistical significance in all final models.

### Reporting Checklist

The Strengthening the Reporting of Observational Studies in Epidemiology (STROBE) [20] checklist was used in the development of the study protocol and will also be followed for the reporting of the final results in the main study paper.

### Ethical Considerations

This study protocol received ethics approval from the ethics committee of the IRCCS Fondazione Don Carlo Gnocchi, Milan, Italy (09_16/12/2022). The study was registered on ClinicalTrials.gov (NCT06758349). The study will be conducted in full accordance with the ethical principles of the Declaration of Helsinki and Italian national regulations. Participation is entirely voluntary. Written informed consent will be obtained from all participants prior to any study procedure. The consent form explicitly details the study aims, procedures, potential risks, benefits, data management practices (including pseudonymization and secure storage), and the right to withdraw at any time without consequence. Participants will be informed that by signing the consent form, they agree to the use of their pseudonymized data for analysis and publication of aggregated results. Participants consented to the publication of aggregated, anonymized data derived from their assessments and questionnaires. In accordance with agreements among all study investigators, the study results and any resulting scientific publications will be disseminated in compliance with applicable regulations and publication standards. It should be noted that, as agreed between all participants in the scientific study, the results and the drafting of the scientific article will be published in compliance with the rules in force.

All information, both personal and clinical, collected during this study is confidential and will be treated in accordance with the relevant regulations. During the research, the following data will be collected: common personal data (first name, surname, and age), lifestyle data (physical activity), professional activity data (eg, instrument played, hours of study or work, and study mode), and health-related data (presence of pain with functional limitation at present or in the past and diagnosis of scoliosis). With the participants’ consent, the data will be entered in pseudonymized form into a computerized REDCap database. Within the digitized database, each patient will be associated with a numeric code (ID) for identification. The key linking participant identities to their corresponding IDs will be stored by the principal investigator on a protected file outside the digitized database for the duration of the study and deleted at the end of the study. Processing operations will be related to research purposes for the conduct of the study. Data will be processed in such a way as to ensure compliance with the principles and requirements established by the EU Regulation 679/2016 and current legislation. Security measures will be implemented to prevent data loss, unauthorized access, unauthorized processing, or any processing that is not consistent with the research purposes described above. Access to and processing of the data will be permitted to the Fondazione Don Carlo Gnocchi’s managers and appointees or authorized persons as well as other personnel involved in the study. Alphanumeric codes will be used to identify the person participating in the study, and only authorized personnel will be able to trace these codes to the participant’s identity. The data collected will be processed and stored by the Foundation for the time necessary to achieve the purposes of the study, after which they will be kept for 10 years from the conclusion of the study itself in the REDCap system, under the responsibility of RMC.

## Results

The study protocol was approved by the ethics committee in November 2022. Data collection initially was scheduled to take place over a 20-month period from May 2023 to December 2024. However, to ensure the achievement of a robust and representative sample size, the recruitment period was extended through April 2026. As of February 2026, a total of 240 participants had been successfully recruited across all participating clinical sites, approaching the predefined target sample size of 250. Preliminary data cleaning and descriptive analyses were initiated in February 2026, with full statistical analysis currently underway. On the basis of the current progress of the study, final results are expected to be available for publication by July 2026.

## Discussion

### Anticipated Findings

This study is designed to provide a comprehensive quantitative analysis of postural deviations in professional orchestral musicians and to explore their association with pain and PPL. By systematically comparing posture during instrumental performance and in a neutral, instrument-free condition, the study aims to identify posture-related associated factors, compensatory strategies, and their potential impact on musicians’ health and professional functioning.

Beyond estimating the prevalence of postural alterations, pain, and PPL, this investigation seeks to clarify how instrument-specific demands and asymmetrical playing positions influence musculoskeletal load. The comparison between neutral sitting posture and instrument-related posture is expected to highlight the extent to which musical performance exacerbates or induces postural deviations, thereby contributing to biomechanical strain. This distinction may help differentiate between baseline postural characteristics and those primarily driven by instrumental practice.

The anticipated findings may reinforce existing evidence that prolonged exposure to forced and asymmetrical postures represents a major contributor to musculoskeletal disorders in musicians. Instruments requiring sustained elevation of the upper limbs, trunk rotation, or unilateral loading—such as bowed string and certain wind instruments—are likely to be associated with greater degrees of postural imbalance, particularly at the cervical, shoulder, and scapular levels. These deviations may correlate with higher rates of upper limb and spinal pain, supporting the hypothesis that compensatory postural behaviors act as a key intermediate mechanism linking instrumental demands to PRMDs.

A central aspect of this study is the investigation of compensatory strategies adopted by musicians to meet technical and artistic performance requirements. While these adaptations may allow musicians to maintain performance quality in the short term, they may also increase cumulative musculoskeletal load over time, predisposing individuals to PRMDs. The identification of such compensatory patterns through objective postural measurements may therefore provide clinically meaningful insights that complement self-reported symptoms and disability assessments.

The use of standardized photographic acquisition and quantitative postural parameters represents a strength of the study, offering an objective framework for posture evaluation in a population that is often assessed primarily through questionnaires. Although the posture analysis device used is not formally validated, its use is consistent with established principles of clinical postural assessment and allows for reproducible measurements across participants. The integration of postural data with validated outcome measures of pain and disability enhances the ecological and clinical relevance of the findings.

Several limitations must be acknowledged. The cross-sectional design does not allow causal relationships between postural alterations, pain, and PPL to be established, and reverse causality cannot be excluded, as pain itself may influence posture. The use of a nonvalidated questionnaire for demographic and musical activity data may introduce reporting bias. Furthermore, although assessments were conducted in real-world orchestra settings to enhance ecological validity, variability in environmental conditions may have influenced postural measurements. Nevertheless, these limitations are offset by the relatively large sample size and the inclusion of professional musicians actively engaged in orchestral performance.

The results of this study are expected to have relevant implications for clinical practice, occupational health, and preventive strategies in the performing arts. By identifying posture-related factors and compensatory mechanisms associated with pain and PPL, the findings may inform the development of targeted ergonomic interventions, including instrument-specific adaptations, posture education, and individualized physiotherapeutic or occupational therapy programs aimed at reducing injury risk and supporting long-term professional sustainability.

The dissemination of the study results will be pursued through multiple complementary channels to maximize scientific, clinical, and professional impact. The primary findings will be submitted for publication in peer-reviewed international journals focusing on occupational health, performing arts medicine, musculoskeletal rehabilitation, and ergonomics. Where appropriate, secondary analyses (eg, instrument-specific or posture-specific findings) may be reported in additional manuscripts.

The results will also be presented at national and international conferences in the fields of occupational medicine, physical and rehabilitation medicine, physiotherapy, ergonomics, and performing arts medicine, fostering interdisciplinary dialogue and knowledge exchange.

To promote knowledge translation, tailored reports and educational materials will be shared with participating orchestras and musicians, highlighting practical implications and preventive recommendations in accessible language. When feasible, workshops or seminars may be organized in collaboration with orchestras to support the implementation of evidence-based ergonomic and preventive strategies.

In addition, the findings may be incorporated into educational initiatives for health care professionals involved in musicians’ health, including physiotherapists, occupational therapists, and physicians. Aggregated and anonymized data may also support future collaborative research projects and inform institutional or occupational health policies related to the performing arts.

### Conclusions

This study aims to advance the understanding of the relationship between posture, instrumental performance, pain, and PPL in professional orchestral musicians. By quantitatively comparing posture with and without the instrument, it seeks to identify postural deviations and compensatory mechanisms associated with pain and PPL.

The expected findings may contribute to the development of evidence-based preventive and ergonomic interventions tailored to the specific demands of different musical instruments. In the long term, this approach has the potential to improve musicians’ health, reduce the burden of PRMDs, and promote professional longevity, supporting a preventive and sustainable model of occupational health in the performing arts.

## Abbreviations

OPErA: Orchestral Posture Ergonomic Assessment
PRMD: playing-related musculoskeletal disorder
REDCap: Research Electronic Data Capture
STROBE: Strengthening the Reporting of Observational Studies in Epidemiology

## References

[1] Sousa CM, Machado JP, Greten HJ, Coimbra D (2017). Playing-related musculoskeletal disorders of professional orchestra musicians from the North of Portugal: comparing string and wind musicians. Acta Med Port.

[2] Zaza C, Charles C, Muszynski A (1998). The meaning of playing-related musculoskeletal disorders to classical musicians. Soc Sci Med.

[3] Kok LM, Huisstede BM, Voorn VM, Schoones JW, Nelissen RG (2016). The occurrence of musculoskeletal complaints among professional musicians: a systematic review. Int Arch Occup Environ Health.

[4] Cruder C, Barbero M, Koufaki P, Soldini E, Gleeson N (2020). Prevalence and associated factors of playing-related musculoskeletal disorders among music students in Europe. Baseline findings from the Risk of Music Students (RISMUS) longitudinal multicentre study. PLoS One.

[5] Gómez-Rodríguez R, Díaz-Pulido B, Gutiérrez-Ortega C, Sánchez-Sánchez B, Torres-Lacomba M (2020). Prevalence, disability and associated factors of playing-related musculoskeletal pain among musicians: a population-based cross-sectional descriptive study. Int J Environ Res Public Health.

[6] Fishbein M, Middlestadt SE, Ottati V, Straus S, Ellis A (1998). Medical problems among ICSOM musicians: overview of a national survey. Med Probl Perform Artists.

[7] Cruder C, Koufaki P, Barbero M, Gleeson N (2019). A longitudinal investigation of the factors associated with increased RISk of playing-related musculoskeletal disorders in MUsic students (RISMUS): a study protocol. BMC Musculoskelet Disord.

[8] Steinmetz A, Scheffer I, Esmer E, Delank KS, Peroz I (2015). Frequency, severity and predictors of playing-related musculoskeletal pain in professional orchestral musicians in Germany. Clin Rheumatol.

[9] Barczyk-Pawelec K, Sipko T, Demczuk-Włodarczyk E, Boczar A (2012). Anterioposterior spinal curvatures and magnitude of asymmetry in the trunk in musicians playing the violin compared with nonmusicians. J Manipulative Physiol Ther.

[10] Kok LM, Vlieland TP, Fiocco M, Nelissen RG (2013). A comparative study on the prevalence of musculoskeletal complaints among musicians and non-musicians. BMC Musculoskelet Disord.

[11] Ramella M, Fronte F, Converti RM (2014). Postural disorders in conservatory students: the Diesis project. Med Probl Perform Art.

[12] Stanhope J, Milanese S (2016). The prevalence and incidence of musculoskeletal symptoms experienced by flautists. Occup Med (Lond).

[13] Bernard BP, National Institute for Occupational Safety and Health (1997). Musculoskeletal Disorders and Workplace Factors: A Critical Review of Epidemiologic Evidence for Work-Related Musculoskeletal Disorders of the Neck, Upper Extremity, and Low Back.

[14] Gómez-Galán M, Callejón-Ferre ÁJ, Pérez-Alonso J, Díaz-Pérez M, Carrillo-Castrillo JA (2020). Musculoskeletal risks: RULA bibliometric review. Int J Environ Res Public Health.

[15] MacDermid JC, Tottenham V (2004). Responsiveness of the disability of the arm, shoulder, and hand (DASH) and patient-rated wrist/hand evaluation (PRWHE) in evaluating change after hand therapy. J Hand Ther.

[16] Gummesson C, Ward MM, Atroshi I (2006). The shortened disabilities of the arm, shoulder and hand questionnaire (QuickDASH): validity and reliability based on responses within the full-length DASH. BMC Musculoskelet Disord.

[17] Chhabra HS, Kapoor KS (2014). New modified English and Hindi Oswestry Disability Index in low back pain patients treated conservatively in Indian population. Asian Spine J.

[18] Vernon H, Mior S (1991). The Neck Disability Index: a study of reliability and validity. J Manipulative Physiol Ther.

[19] Fortin C, Feldman DE, Cheriet F, Labelle H (2011). Clinical methods for quantifying body segment posture: a literature review. Disabil Rehabil.

[20] von Elm E, Altman DG, Egger M (2007). The Strengthening the Reporting of Observational Studies in Epidemiology (STROBE) statement: guidelines for reporting observational studies. PLoS Med.

